# Impact of quality and transparency in scientific writing on the reduction of animal usage in experimental protocols: a review based in pertinent literature

**DOI:** 10.3389/fvets.2024.1394113

**Published:** 2024-05-30

**Authors:** Matheus M. Neves, Sandra G. Klein, Ray C. Silva, Lucas M. M. Bernardes, Serena M. Malta, Thiago N. Vieira, Rafael B. Rosa, Isabela L. Lima, Flávia B. Ferreira, Murilo V. Silva

**Affiliations:** ^1^Biotechnology in Experimental Models Laboratory - LABME, Federal University of Uberlândia, Uberlândia, Brazil; ^2^Institute of Biotechnology, Federal University of Uberlândia, Uberlândia, Brazil; ^3^Rodents Animal Facilities Complex, Federal University of Uberlandia, Uberlândia, Brazil

**Keywords:** laboratory animal science, data reproducibility, 3R’s, scientific writing, animal models

## Abstract

The irreproducibility in scientific research has become a critical issue. Despite the essential role of rigorous methodology in constructing a scientific article, more than half of publications, on average, are considered non-reproducible. The implications of this irreproducibility extend to reliability problems, hindering progress in technological production and resulting in substantial financial losses. In the context of laboratory animal research, this work emphasizes the importance of choosing an appropriate experimental model within the 3R’s principle (Refine, Reduce, Replace). This study specifically addresses a deficiency in data specification in scientific articles, revealing inadequacies in the description of crucial details, such as environmental conditions, diet, and experimental procedures. For this purpose, 124 articles from journals with relevant impact factors were analyzed, conducting a survey of data considered important for the reproducibility of studies. Important flaws in the presentation of data were identified in most of the articles evaluated. The results of this study highlight the need to improve the description of essential information, standardizing studies, and ensuring the reproducibility of experiments in areas such as metabolism, immunity, hormones, stress, among others, to enhance the reliability and reproduction of experimental results, aligning with international guidelines such as ARRIVE and PREPARE.

## 1 Introduction

The construction of scientific thought involves the necessity of a methodology, which must be well-structured in the written text so that the new knowledge generated can be reliable and well-founded, and, if necessary, can be revised as a way of generating even more information (1). Therefore, under the stipulated conditions in the construction of a scientific article and using the appropriate methods, it is possible to arrive at the same results and conclusions as those that were previously done and shared.

However, this is not the reality of contemporary research, according to a survey conducted by the journal Nature (2), which considered the experiences of more than 1,500 researchers. On average, more than half of the publications are not reproducible under the described methodology. Thus, the foundations for the trust and accuracy of the results produced in the light of scientific knowledge are often called into question, due to confusion and a lack of guidance in scientific writing.

In this context, irreproducibility in different areas of science causes a series of problems, ranging from the unreliability of the produced results, which hinders progress in technological production in various areas, to enormous financial expenditure without justifiable returns. In the biomedical research field, where experimental animal models are crucial, no different information is expected. For example, an annual loss of 28 billion dollars in biomedical research without fruitful outcomes due to this irreproducibility (3).

When considering the life sciences, research on drugs, treatments for diseases, and understanding pathologies, the confidence in the results is essential for the advancement of the quality of life and health of humanity. However, like other areas, there are various obstacles to reproducibility which becomes even more significant when considering the use of animals as experimental models (4). This reality can occur due to various factors, such as bias in research construction, as well as problems in writing and data selection (5) and poor experimental design (6–9).

In the scope of animal research, the choice of an appropriate experimental model ensures an important step in the experimental design of studies that seek to understand the physiological, anatomical, and genetic functioning of a specific topic in relation to what occurs on a biological scale and can possibly be extended to the human context (5). Furthermore, it is important that experimental protocols are well described in works published in scientific journals. This practice significantly impacts the reproducibility of data, as it standardizes protocols and makes documentation on these procedures more robust (2) It has already been raised that the omission of data has contributed to the reproducibility crisis (3, 10).

Within the framework of the 3R principle (Refine, Reduce, Replace) proposed by Russell and Burch, experiments involving animals should be replaced with alternatives whenever possible. However, if an animal experiment cannot be replaced, the number of animals should be limited, and procedures should be refined to minimize the pain, suffering, and distress caused to the animals by the experiment (11).

Considering the data deficiency situation in scientific articles, our group has been seeking to understand the reasons behind such a crisis. This discussion proves to be of great importance for the improvement of science because it will present a reflection on how researchers sometimes may contribute to this reproducibility crisis. Not just because the experiments are flawed, but at the time of writing the article data, they do so inadequately. Our results can lead to positive outcomes, such as the non-use of animals when not necessary, as there is already access to previously described data That said, this present work aims to analyze how scientific articles report relevant information involving animal models and how their description can contribute to limited reproducibility. For this purpose, 124 articles from different journals with considered relevant impact factors were evaluated. Data presented in each article, such as approval by the Institutional Animal Care and Use Committee (CEUA), brand of feed, environmental factors, anesthesia, analgesia, and statistical data, were surveyed.

In this review, we synthesize information from various studies pertinent to laboratory animal science, discussing the main topics that must be considered in the writing and planning of a scientific paper inserted in these subject areas in order to make it robust, reliable, reproducible, and to provide an ethical scientific context that adheres to recommendations for animal care and experimentation, prioritizing the health of both the animals and the researcher.

Within the literature, significant studies showcase how the composition of a scientific article influences scientific reproducibility. Therefore, as a distinctive feature, we aim to illustrate how the comprehensive description of the model and its associated aspects impact not only reproducibility but also the ethics of animal usage.

Accordingly, our review aims to address essential aspects in the science of laboratory animal research and how their detailed description in studies can contribute to more reliable science.

## 2 Methodology

The paper selection for this review followed stringent criteria to ensure the inclusion of relevant and impactful studies. Specifically, peer reviewed and relevant in the biomedical field, published between 2015 and 2020 with a journal impact factor surpassing 1.0 were considered. The impact factor calculation used a chrome extension named “PubMed Impact Factor” and considered the Journal Citation Reports (JCR) Quartile, with inclusion limited to papers falling within Quartiles Q1 to Q4. Inclusion criteria also involved the presence of the keyword “animal model” and “mice” and “experimental models” to focus on studies directly related to animal models. English-language papers were exclusively chosen, and the selection prioritized studies involving animals other than humans. To maintain thematic integrity, review papers were excluded, as were studies that did not incorporate animal models.

The selection was made based on the following research framework in PubMed database: “((“2015/01/01” [Date - Publication]: “(((“2015/01/01”[Date - Publication]: “2020/01 / 01”[Date - Publication])) AND (animal model[Text Word])) AND (mice[Text Word])” and an Impact Factor filter. In this research, 250 articles were randomly selected for analysis, and upon applying the inclusion and exclusion criteria, 124 articles remained.

The articles we consider encompass the following experimental models: mouse, rat, *Drosophila melanogaster*, *Aelosoma viride*, Eurasian blue tit, cattle, dragon lizard, *Lampronycteris brachyotis*, *Micronycteris megalotis*, *M. microtis*, *M. homezi*, *M. minuta*, and rabbit. All the studies used are from the biomedical field or related to biological sciences.

## 3 The contribution of transparency in article writing to the ethical use of animals

As described above a total of 124 articles from various journals, with a considered significant impact factor (above 1.0), were examined (articles available at Supplementary information S1). An analysis of the data presented in each article was conducted, encompassing presence of statistical methods for group formation, the brand of feed used, environmental variables, anesthesia, and analgesia procedures, as well as statistical data.

**Figure 1:**
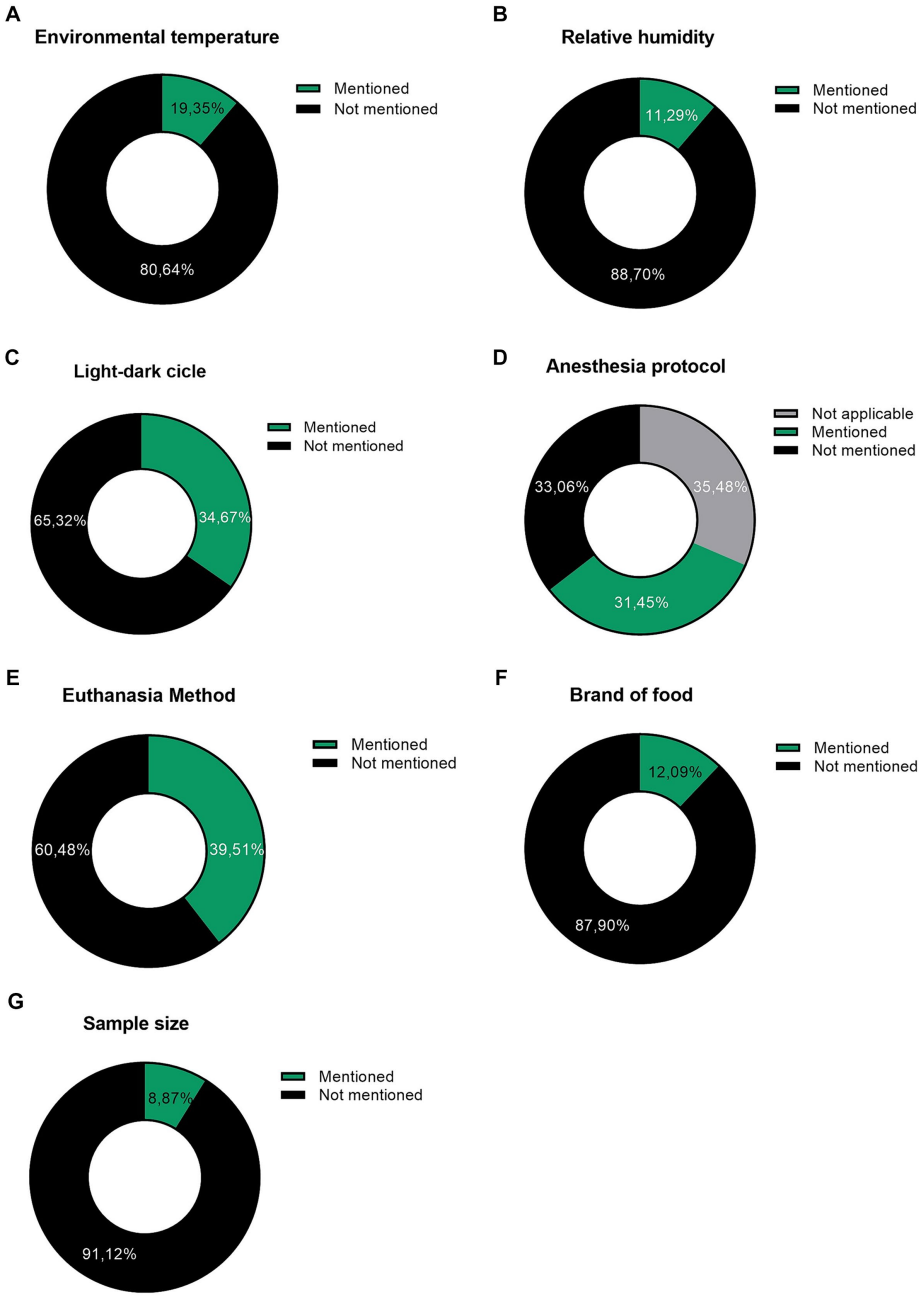
Graphs representing the items evaluated in the study articles. Environmental factors in animal facilities: **(A)** Environmental temperature. **(B)** Air humidity and **(C)** Light–dark cycle; Experimental procedures: **(D)** Anesthesia protocol and **(E)** Euthanasia method; Food: **(F)** Brand of food. **(G)**: Sample size calculation formula. The articles were evaluated for the presence of information, whether it was mentioned and whether it contained details. Despite being basic items, in most articles they are not mentioned. Data were expressed as a percentage, where 100% indicates a total of 124 articles.

The results revealed a considerable number of factors with unsatisfactory information, significantly contributing to the lack of reproducibility in assays. For instance, only 12.9% of the articles mention the brand of food used. Over half of the articles fail to specify crucial details, such as brightness, temperature, and humidity to which the animals were subjected. Additionally, 60.5% of the articles do not describe the euthanasia method employed, and 91.1% do not present the calculation formula of the sample size, among other elements that can directly impact the reproducibility of the research (Figure 1).

Handling laboratory animals requires rigorous data control. All the data presented in this study are directly related to the health, well-being, and immunity of the animals. Depending on the study to be conducted, this data becomes extremely important. Any changes in the photoperiod can lead to hormonal alterations (12), impacting the results. Nutritional or environmental changes can alter factors related to immunity (13), once again causing a negative impact on the reproducibility of desired results.

Thus, by highlighting these shortcomings in data specification, the need to improve the description of essential information (diet, environmental factors, pain-related factors, among others) becomes evident. With the deficit of these details, experiments that would not need to be repeated may be conducted again, contradicting the principles proposed by the 3R’s (14) and the sustainable development goals outlined by the UN (15).

To prevent this, scientific journals and platforms should require the complete disclosure of data from experimental models, preferably following international regulations such as the ARRIVE guideline or the PREPARE guideline (16).

The goals of sustainable development are linked to animal welfare. Animals in a state of well-being are more productive and yield products of higher quality. Similarly, research animals generate more reliable results and foster more promising technologies and innovations. Furthermore, more effective public policies from veterinary bodies and responsible entities can guide and implement positive actions for animal welfare (15).

To this end, there are some published guidelines, such as ARRIVE and PREPARE, which can contribute to animal welfare and the refinement of research. The guidelines encourage researchers to report on randomization, blinding, sample size calculations, management and procedures, welfare monitoring, euthanasia, among others. Thus, under firm convictions about the importance of these issues and supported by evidence from other areas of research, there is a current consensus that scientists should adopt these practices whenever possible to produce work with greater impact and applicability (16).

## 4 The importance of experimental models for the development of science, technology, and innovation

Vertebrate animals have been used as models of anatomy and physiology since the beginning, where there are records of Greek doctors who dissected animals for anatomical studies (17). In the 17th century, the moral questions surrounding the use of animals began to be raised and between the 19th and 20th centuries, the pharmacopoeia included effective and scientifically tested medicines, which led to a greater understanding of the importance and validity of animal-based research (18, 19).

Currently, animal models are essential for several fields within biomedical research, such as cancer, neuroscience, pharmacology and toxicology, neurobiology of diseases, endocrinology, public health, palliative medicine, discovery and testing of new medicines, vaccines and other biological products whose validation requires preclinical animal studies (19). Its use is based on the principle of replicating physiological and pathological processes, with the species selected according to the objective and hypothesis of each project (20).

In recent years, for example, different animal species have been used to study the 2019 Coronavirus pandemic. Through murine, primate, porcine and even zebrafish models, neurological, behavioral, cardiovascular, and oncological disorders can be studied as they are also new therapeutic approaches are being developed. Recently, nematodes and arthropods are some of the new alternatives (21). Today, the majority of species used in biomedical research are rodents, as they are considered ideal models for studying pathologies that affect human populations due to their physiological homology (21).

We can reflect the importance of using animals by observing the number of important studies, such as those for the Nobel Prize in Physiology and Medicine, in which 90% of them used animals (22). In 2005, a global survey was carried out, estimating the number of animals used (23). This estimate showed the use of 58.3 million animals in 179 countries. This same group made an estimate for 2015, which was around 79.9 million animals, an increase of 36.9% compared to 2005 (24). The Mutual Society (not-for-profit organization) “Understanding Animal Research” has data from 2020 and recorded that the European Union used 8,624,692 animals, with 91% of the animals used for experimental purposes being mice, fish, rats, and birds, while cats, dogs and primates represented 0.2%. In the USA, unofficial estimates that include mice, rats and non-mammalian vertebrates estimated the use of 12 to 24 million animals. In Canada, the Canadian Council on Animal Care (CCAC) reported that 5,067,778 animals were used in 2020. Therefore, we can conclude that many studies around the world use animals, reaffirming their importance in science (25).

Currently, mice are the most used in human biology research (26), among all animals used in research, mice account for almost 60% of the total (27). This is due to their genetic and physiological similarity with humans, short gestation times, genetically homologous inbred strains, easy handling and easy maintenance (26).

## 5 Ethical and legal aspects in the use of animals and 3R’s headings

Animal experimentation has, for an extended duration, been surrounded by a series of inquiries, both from the scientists conducting it and from the general population, questioning the obtained results at the expense of animal lives. In this context, an array of thoughts concerning animal well-being, ethics, and care have evolved over time. Presently, all these concepts are grounded in a set of three principles established by Russell and Burch in 1959 (11).

The guidelines are founded on the concepts of Reduction, Refinement, and Replacement, which establish ethical and legal concepts globally for the use of animals in re-search, imbuing a humanitarian perspective, dignity, and protection against suffering, pain, and stress upon these animals (1). It is vital to emphasize that, based on these principles, the entire legal and ethical framework of animal experimentation must be based on shaping the concept of what is dignified in the lives of animals, elevating them to a level of rights equivalent to humans. This principle, as advocated since 1973 and enshrined in the Swiss Constitution in 1992 and addressed by Bolliger ensures their moral standing (28).

In this context, several other countries have incorporated the idea of animals as beings with their consciousness, rights, and moral reality into their highest legislation, such as India, Brazil, Slovenia, Germany, Luxembourg, Austria, Egypt, in addition to the guidelines of the European Union and the United States. This shift in perspective considers animals as subjects with their rights and moral standing rather than mere property and objects, as they were often viewed (29). Consequently, limitations are set on how animal life can be manipulated and used in experiments, with specific barriers concerning sensations and intrinsic well-being guarantees.

It is worth emphasizing that the concept of dignity, and therefore the right not to suffer and to have one’s needs met, which was once the exclusive domain of human beings, a concept upheld for a considerable period and reinforced by authors such as Giovanni Pico della Mirandola (30) and Immanuel Kant (31) has been extended to all animals. This is due to their sentience akin to that of humans, and their entitlement to the same rights, which ensures both ethical and legal legitimacy to the science produced with their assistance (32).

Furthermore, it is crucial to consider that research conducted without ethical considerations when it comes to animal use creates an experimental environment where the produced results cannot be relied upon. Ethical treatment implies the construction of well-being, ensuring the expression of phenotypes without alterations caused by stress (33). Thus, experimental reproducibility, a contemporary topic, especially when dealing with the use of model organisms, is only achievable when measures are taken, such as proper handling, anesthesia protocols, and stress avoidance, guaranteeing the veracity of the results attained in science (1).

It is important to emphasize that the new guidelines regarding the care of animal testing and experiments are primarily centered on those established in Directive 2010/63/EU by the European Union in 2010. This directive presented, suggested, and encouraged other legislations to adopt similar measures, a call heeded by countries across all continents. These regulations were constructed and based, once again, on the concept of dignity. The idea is that a legal and punitive framework can only be established for those who disregard it when there is a set of principles defining what is dignified and guaranteed for animal life. It asserts that the right of animals, being lives that should not be treated as mere possessions for utilitarian purposes, must be recognized. Instead, animals should be regarded as valuable contributors, with a significance equal to that of the researcher’s existence, in the pursuit of scientific progress (34).

## 6 Important environmental factors in experimentation with rodents

Environmental factors are the set of variables that constitute the environment in which the animal lives, including physical, social and management aspects. In animal facilities, these factors are determined and monitored by man, since the environment is controlled. In this section we will emphasize the importance of detailing the housing and care factors of animals used in research to ensure experimental reproducibility, thereby facilitating a reduction in their use in subsequent studies. For illustration, we will exemplify physical factors, such as temperature, humidity, and luminosity (35).

Temperature is one of the first and most basic variables that must be observed when thinking about ambience. Animals housed outside their thermoneutral zone will have important physiological changes such as changes in metabolism, blood pressure, sleep, and rest time, circulating immune cells, among others (36). The most common in rat and mouse laboratories is housing below the thermoneutral zone, around 22°C, when the ideal would be around 30°C. This is mainly due to human thermal comfort, which is affected in these working conditions together with the use of personal protective equipment and activities carried out in animal facilities (36, 37).

To overcome this situation, some strategies can be used, such as maintaining an average temperature that does not affect animals or humans so much, associated with this, offering environmental enrichments that contribute to thermal insulation, such as materials for nesting and shelters and, whenever as possible, keep the animals in groups, so that they warm each other (38). In cases where the animal’s thermoneutral temperature is lower than that of humans, environmental enrichment strategies with water, ice and ventilation may be useful.

The transparency in information regarding temperature is of paramount importance in the ability of a study to be reproducible without animal experimentation. This is because it allows for the standardization of a factor that directly influences animal behavior, in addition to their physiological and immunological functioning. This transparency enables the prediction of deviations in results across different repetitions due to variations in the temperature to which the animal is subjected.

As for relative air humidity, it is essential to guarantee the well-being and health of animals, as many species are sensitive to environmental variations. Humidity is directly related to thermal sensation as it can facilitate or impair gas exchange depending on the ambient temperature. In addition, air humidity much lower than recommended can lead to irritation of the airways and greater susceptibility to diseases such as Influenza (39). Therefore, careful monitoring and maintenance of air humidity in animal facilities is essential to ensure ideal breeding and experimentation conditions, promoting reliable and ethical results in scientific studies (35).

The same holds true for humidity in relation to temperature. When the humidity value is standardized between the reference work and the one being developed, the conditions, particularly pertaining to respiratory capacity and the animal’s susceptibility to infections, become normalized. This is a crucial factor in some research studies, making it necessary to report these conditions in the animal housing section.

Light plays a significant role in animal experimentation, influencing both welfare and the scientific results obtained. Animals, such as laboratory rodents, are sensitive to light and dark cycles, and careful manipulation of these patterns is essential to maintain normal physiology and behavior (40). In addition to providing an adequate light source, regularity in light–dark cycles is crucial to preserving the animals’ circadian rhythms, impacting important variables such as insulin resistance, gut microbiota dysregulation, sleep patterns and response to external stimuli (41). Controlled lighting also plays a role in minimizing stress in animals by promoting a more stable and predictable environment (42). Therefore, attention to luminosity is essential to ensure the validity and replicability of studies, while also considering the ethical impact and welfare of the animals involved in the experiment (43).

Finally, regarding the examples provided on how housing factors can alter the quality and reproducibility of an experiment involving animals, light also plays a significant role. It is essential to be transparent about this data because it not only influences factors such as normal behavior, reproductive capacity, microbiota, among others, but when presented excessively, it can cause direct harm to animal health, such as issues in the retina, hindering the faithful reproduction of a study (44).

Certainly, careful consideration of environmental factors such as temperature, humidity and light are essential to ensure reliable and ethical results in animal experimentation (43). It is worth noting that the information provided in this section is not the only important aspect to consider in terms of transparency when writing a scientific paper and ensuring its reproducibility, especially in experiments involving animals. These examples are part of a much broader range that encompasses aspects such as analgesia and anesthesia methodologies, housing space and equipment, as well as statistical methods for forming groups with exclusion and inclusion criteria.

**Figure 2:**
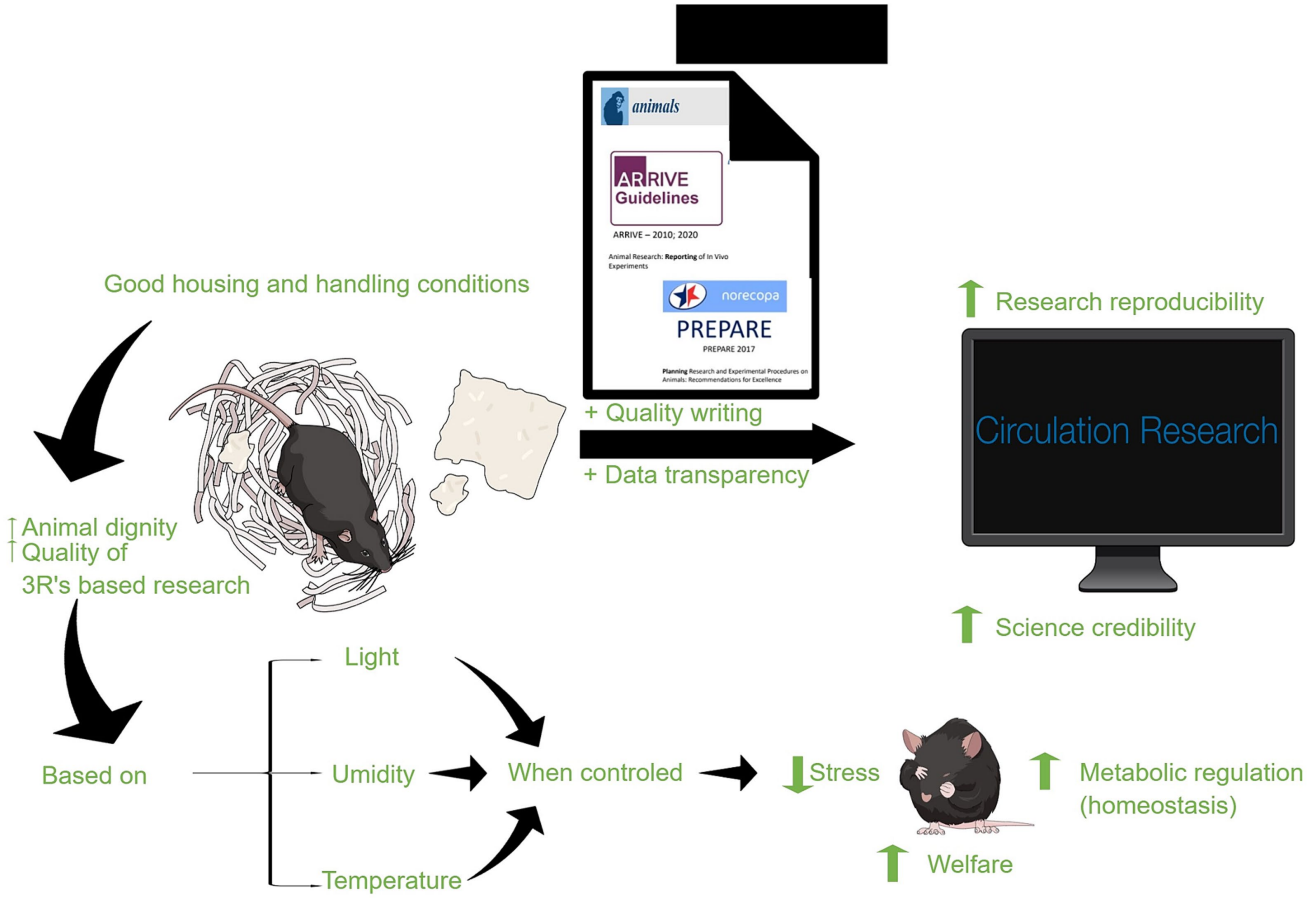
Factors that influence animal well-being and provide favorable conditions for their feeding, natural behavior, and self-care, leading to an increase in data confidence and reproducibility.

Other important criteria in the design of an experiment involving animal models should be guided, from its initial conception, by specific concepts and guidelines for reproducibility and experimental reliability. In this work, we will mention the ARRIVE and PREPARE guidelines. By recording these variables and understanding their direct influence on the well-being of laboratory animals, researchers can improve the validity and relevance of their studies. The search for conditions that mimic the animals’ natural environment, combined with practices that promote their comfort, not only improves the integrity of experimental data, but also reinforces fundamental ethical principles (45) (see Figure 2).

## 7 ARRIVE and PREPARE guidelines

In contemporary times, with the advancement of biomedical research utilizing animals as experimental models, it has become essential to establish standards and guidelines for writing to emphasize key points for constructing transparent, comprehensible, and reproducible scientific papers. The first of these guidelines is known as ARRIVE (Animals in Research: Reporting *In Vivo* Experiments) (46). Proposed in 2010, it is directly related to a set of older recommendations from 1996, CONSORT (Consolidated Standards of Reporting Trials) (47). These guidelines were constructed based on systematic reviews and scientific research, along with input from animal experimentation experts worldwide. The goal was to identify, within the scientific scope, the necessary information to enhance the quality of work involving such models.

ARRIVE should not only be considered during experiments but throughout the entire thought, planning, and writing process related to the work. These aspects were well described in the original article suggesting this guideline, considering the quality of statistical planning and the presentation of data. It also suggests sharing details such as the physical characteristics of animal housing, demonstrating respect for animal dignity by the authors (46). Building upon ARRIVE, a set of enhancements was proposed in 2020, aiming to maximize the functionality and success of this directive. This resulted in the creation of ARRIVE 2.0, offering a more recent perspective and updating concepts derived from advances in scientific research. The objective is to increase adherence by both researchers and journals (48).

In this context, to complement the gaps addressed by ARRIVE and to synthesize the recommendations of the 2010/63 directive of the European Union (49), another set of guidelines was created: PREPARE (Planning Research and Experimental Procedures on Animals: Recommendations for Excellence). Introduced in 2017, PREPARE is designed to be followed throughout the entire research and scientific writing process. It delves into ethics, proper care, respect for animal dignity, the researcher’s relationship with the research institution, and animal care. PREPARE encourages transparency in presenting data and methodologies related to necropsies, sanitary and genetic monitoring, legal aspects, and detailed experimental procedures (50).

## 8 Discussion

The need to reduce the number of animals in research is based on several reasons of an ethical, scientific, economic, and social nature. In this paper we present data that point out ways to reduce the number of animals in experimental protocols at no cost, bringing up crucial points that are not presented in detail in many articles we evaluated. We show a reflection that will contribute to improving all the aspects presented above, and to improving the quality of life of professionals who work with laboratory animals. Currently, many professionals suffer from compassion fatigue, also known as emotional exhaustion, which is a psychological phenomenon that can affect veterinarians and laboratory animals technicians involved in animal experimentation due to constant contact with the suffering of animals (51).

In this article, we provide a specific insight into how the correct and transparent writing of a study involving research with laboratory animals serves as a means to share data that can lead to a reduction in the number of animals used. This is due to the possibility of faithfully reproducing experimental conditions, thereby eliminating the need for unnecessary repetitions and uses of large quantities of animals. Thus, in addition to all the other benefits already mentioned arising from quality writing, the major contributors to compassion fatigue, such as excessive euthanasia and continuous, repetitive exposure to protocols that induce animal welfare issues and desensitization to suffering, would be reduced and avoided (52). This fosters a healthier ecosystem for work and research.

The results obtained in this study highlight the importance of a thorough description of relevant data and information in articles. The need to address aspects such as environmental condition, pain control, and welfare improving methods. This approach not only contributes to the standardization of studies but also provides essential insights into metabolism, immunity, hormonal factors, and stress—fundamental components for ensuring the reproducibility of scientific assays, and to create a respectful and ethical research environment, both for researchers and for animals, these who are cornerstones for the advancement of science.

By emphasizing the significance of these elements, researchers can enhance the quality and reliability of their studies, fostering a more solid foundation for future research and scientific advancements. The inclusion of these critical details not only benefits the comprehensive understanding of experiments but also facilitates replication by other scientists, thereby strengthening the validity and robustness of the obtained results, in addition to reducing the need for unnecessary repetitions of experimental protocols and greater exposure to suffering and isolation by researchers, it decreases the possibility of compassion fatigue and prioritizes their mental health.

In summary, careful attention to specific aspects in the description of data in scientific articles not only addresses the demands of the academic community but also significantly contributes to the progression of science.

## Author contributions

MN: Writing – review & editing. SK: Writing – original draft. RS: Writing – original draft. LB: Writing – original draft. SM: Writing – original draft. TV: Writing – original draft. RR: Writing – original draft. IL: Writing – original draft. FF: Writing – original draft. MS: Writing – review & editing, Writing – original draft.
